# Chronic HDV Infection Shows Higher HBsAg Isoform Levels than HBV Infection, Paralleling HDV Replicative Activity

**DOI:** 10.3390/v18050515

**Published:** 2026-04-30

**Authors:** Stefano D’Anna, Lorenzo Piermatteo, Alessia Magnapera, Ilaria Grossi, Caterina Tramontozzi, Antonella Olivero, Leonardo Duca, Giulia Torre, Elisabetta Teti, Andrea Di Lorenzo, Vincenzo Malagnino, Marco Iannetta, Sandro Grelli, Pierpaolo Paba, Ada Bertoli, Francesca Ceccherini-Silberstein, Leonardo Baiocchi, Simona Francioso, Ilaria Lenci, Michele Milella, Annalisa Saracino, Alessia Ciancio, Giuseppina Brancaccio, Loredana Sarmati, Pietro Lampertico, Mario Rizzetto, Gian Paolo Caviglia, Romina Salpini, Valentina Svicher

**Affiliations:** 1 Department of Biology, University of Rome Tor Vergata, 00133 Rome, Italy; stefanodanna26@gmail.com (S.D.); lorenzo.piermatteo@uniroma2.it (L.P.); alessiamagnapera97@gmail.com (A.M.); ilariagrossi8@gmail.com (I.G.); c.tramontozzi1@gmail.com (C.T.); giuliatorre.1997@gmail.com (G.T.); 2 Department of Medical Sciences, University of Turin, 10126 Turin, Italy; antonella.olivero@unito.it (A.O.); alessia.ciancio@unito.it (A.C.); mario.rizzetto@unito.it (M.R.); gianpaolo.caviglia@unito.it (G.P.C.); 3 Department of Experimental Medicine, University of Rome Tor Vergata, 00133 Rome, Italy; duca.leonardo@hotmail.it (L.D.); grelli@med.uniroma2.it (S.G.); bertoli@uniroma2.it (A.B.); ceccherini@med.uniroma2.it (F.C.-S.); 4 Department of Systems Medicine, Infectious Disease Clinic, Tor Vergata University, 00133 Rome, Italy; elisabetta.teti@gmail.com (E.T.); andrea.dilo23@gmail.com (A.D.L.); malagninovincenzo@gmail.com (V.M.); marco.iannetta@uniroma2.it (M.I.); sarmati@med.uniroma2.it (L.S.); 5 Virology Unit, Policlinico Tor Vergata, 00133 Rome, Italy; pierpaolo.paba@ptvonline.it; 6 Hepatology Unit, Policlinico Tor Vergata, 00133 Rome, Italy; baiocchi@uniroma2.it (L.B.); simona.francioso@uniroma2.it (S.F.); ilaria.lenci@uniroma2.it (I.L.); 7 Department of Biomedical Sciences and Human Oncology, Clinic of Infectious Diseases, University of Bari “Aldo Moro”, 70121 Bari, Italy; michele.milella@tin.it (M.M.); annalisasaracino@gmail.com (A.S.); 8 Department of Life Sciences, Health and Health Professions, Università degli Studi di Roma Link Campus University, 00165 Rome, Italy; g.brancaccio@unilink.it; 9 Division of Gastroenterology and Hepatology, Foundation IRCCS Ca’ Granda Ospedale Maggiore Policlinico, 20122 Milan, Italy; pietro.lampertico@unimi.it; 10 CRC “A. M. and A. Migliavacca” Center for Liver Disease, Department of Pathophysiology and Transplantation, University of Milan, 20122 Milan, Italy

**Keywords:** chronic hepatitis D, total HBsAg, HBsAg isoforms, HBcrAg, HDV-RNA

## Abstract

Background & Aim: The entry of Hepatitis D Virus (HDV) depends on HBV surface proteins (HBsAg) composed of three isoforms: large-, middle, and small HBsAg. Here, we investigate the levels of total HBsAg and HBsAg isoforms and their correlations with HDV-RNA, HBcrAg, and transaminases in the setting of untreated chronic hepatitis D (CHD). Methods: This study includes 316 HBeAg-negative patients: 192 CHD and 124 with chronic hepatitis B (CHB) as a control group. HBsAg isoforms were quantified by ad hoc-designed ELISAs. Results: The composition of HBsAg isoforms varied between the two groups of patients, with remarkably higher small HBsAg, middle-HBsAg, and large HBsAg in CHD than in CHB. This data was confirmed by multivariable analysis (*p* < 0.0001). Among CHD, HBsAg isoforms positively correlated with HDV-RNA (*p* < 0.0001) and HBcrAg (*p* < 0.0001) but not with HBV-DNA. The results were confirmed by stratifying patients according to HDV-RNA (< or >1000 IU/mL) and HBcrAg (< or >3 logU/mL). Furthermore, CHD patients with ALT > upper limit of normal presented significantly higher S-HBsAg and M-HBsAg levels. Conclusions: CHD is characterized by a more elevated HBsAg isoform production, paralleling HDV-RNA and HBcrAg release. This may suggest a preferential recruitment of HBsAg isoforms in HDV virions at the expense of HBV virions. The association of HBsAg isoforms with higher ALT also suggests their potential contribution in supporting HDV-induced pro-inflammatory stimuli.

## 1. Introduction

Hepatitis D Virus (HDV) is a satellite virus that can infect only in the presence of hepatitis B virus (HBV) since it exploits the HBV surface proteins (collectively defined as hepatitis B surface antigen [HBsAg]) for viral morphogenesis and entry into hepatocytes [1].

HBsAg consists of three proteins: (i) large HBsAg (L-HBsAg) containing the preS-1, preS-2, and S regions, (ii) middle HBsAg (M-HBsAg) containing the pre-2 and S regions, and (iii) small HBsAg (S-HBsAg) containing only the S region [2]. S-HBsAg is the predominant form with major epitopes eliciting an immune response, while L-HBsAg ensures viral entry into the hepatocytes by binding to the sodium-dependent taurocholate co-transporting polypeptide [3]. Although the role of M-HBsAg remains elusive, recent studies have suggested its modulatory effect on the morphogenesis of virions and on immune responses [4,5].

The three HBsAg proteins share the C-terminal S region, representing the target of the commercially available assays used for HBsAg quantification. Thus, these assays permit the quantification of the total amount of HBsAg without distinguishing the contribution of each isoform [6]. Recently, novel assays capable of quantifying HBsAg isoforms have been developed and mainly applied in the setting of chronic HBV infection [7,8,9]. Conversely, a paucity of information is available on the role of HBsAg isoforms in the setting of chronic HDV hepatitis (CHD). The first evidence supports that the composition of HBsAg isoforms may play a role in predicting clinical outcome of CHD and response to the anti-HDV drugs [10,11].

Beyond HBsAg isoforms, other biomarkers are under evaluation. Among them, hepatitis B core-related antigen (HBcrAg) is a composite biomarker that quantifies HBV capsid protein, HBeAg, and the 22 kDa precore protein. Previous findings showed that HBcrAg is detectable, even at high levels, in most patients with CHD despite low HBV-DNA levels [12]. No information is available so far on the correlation of HBcrAg and HBsAg isoforms in patients with CHD.

In this light, this study aims at (i) unravelling the levels of total HBsAg and the composition of HBsAg isoforms in patients with CHD compared to HBV chronically infected patients and (ii) investigating the correlation of HBsAg isoforms with virological and biochemical parameters in the setting of CHD.

## 2. Materials and Methods

### 2.1. Study Population

This cross-sectional study included 192 HBeAg-negative patients with CHD (naïve to bulevirtide), followed in four clinical centers in Italy: Fondazione IRCCS Ca’ Granda Ospedale Maggiore Policlinico of Milan; Policlinico Tor Vergata of Rome; “S. Giovanni Battista” Hospital of Turin Molinette; and Azienda Ospedaliera Universitaria Consorziale Policlinico of Bari. CHD was defined as the persistence of positivity to HBsAg and anti-HDV antibodies for at least 6 months with detectable HDV-RNA [13].

A cohort of 124 patients with HBeAg-negative chronic hepatitis B (CHB), with comparable demographics and rates of NUC treatment, was included as a control group to compare HBsAg isoform levels.

The following information was collected: patients’ demographics, NUC treatment and duration, alanine–aminotransferase (ALT) and aspartate–aminotransferase (AST), platelet count, fibrosis/cirrhosis/hepatocellular carcinoma status, HBV-DNA, HDV-RNA, and total HBsAg. HBV-DNA and total HBsAg were quantified in the centers involved in the study using the following commercial assays: the COBAS/AmpliPrepCOBAS TaqMan HBV assay on the Cobas 6800 system (F. Hoffmann-La Roche Ltd., Basel, Switzerland) for HBV-DNA, and the ARCHITECT-QT assay (Abbott Diagnostics, Santa Clara, CA, USA) or Elecsys^®^ HBsAg II quant II (F. Hoffmann-La Roche Ltd., Switzerland) for total HBsAg. HDV-RNA was quantified by the RoboGene HDV-RNA Quantification Kit 2.0 (Roboscreen Diagnostics, Leipzig, Germany) in 3 centers and by an in-house protocol in the remaining one [14]. Severe fibrosis/cirrhosis was diagnosed if liver stiffness measurement was ≥9 kPa in patients with normal ALT, or ≥12 kPa in patients with ALT ≤ 5x upper limit of normality (ULN), when available, by liver biopsy (Metavir score ≥ F4 or Ishak score ≥ 5) and/or by clinical signs (varices, ascites, encephalopathy) [15].

All patients’ information was treated in a confidential manner, and all clinical data were collected anonymously in an ad hoc-designed database.

### 2.2. Quantification of HBsAg Isoforms and HBcrAg

The levels of the three different HBsAg isoforms (L-, M-, and S-HBsAg) were quantified using three different ad hoc-designed ELISA assays developed in collaboration with Beacle Incorporation (Kyoto, Japan) as described in [9,11]. These assays have been demonstrated to have high sensitivity (detection limit for each protein is 0.1 ng/mL) and a high specificity, based on a sandwich system in which anti-PreS1, anti-PreS2, and anti-S antibodies are used, respectively. As certified by the company, when measuring a specific concentration of antigen using a standard antigen, the percentage of intra-run coefficient of variation (CV) is less than 10%, and the percentage of inter-run CV is less than 15%. First, total serum HBsAg was quantified using the HBs S Antigen Quantitative ELISA Kit, Rapid-II (defined in the formula below as S assay) (Beacle Inc., Kyoto, Japan), by targeting the S region, common to all the HBsAg isoforms.

Subsequently, the HBs pre-S2 Antigen Quantitative ELISA Kit, Rapid (Beacle Inc., Japan), was used to quantify the L- and M-HBsAg, targeting the pre-S2 region (defined as pre-S2 assay). Lastly, the L-HBsAg was quantified by the HBs pre-S1 Antigen Quantitative ELISA Kit, Rapid-II (Beacle Inc., Japan), targeting the pre-S1 region (defined as pre-S1 assay). For all assays, the experimental procedure was carried out according to the manufacturer’s instructions.

The following formulas were applied in order to obtain the levels of:

S-HBsAg = (quantification by S assay) − (quantification by pre-S2 assay);

M-HBsAg = (quantification by pre-S2 assay) − (quantification by pre-S1 assay).

For each patient, total HBsAg, L-HBsAg, M-HBsAg, and S-HBsAg levels were quantified in duplicate.

In all our experiments, the coefficient of determination (R2) of the standard curve was always more than 0.99.

HBcrAg levels were quantified by Fujirebio (LLoQ = 3 logU/mL) in a subset of 96 patients with CHD.

### 2.3. HDV and HBV Sequencing

HDV sequencing of the full genome was performed by the Illumina platform, while HBV sequencing of the HBsAg/reverse transcriptase genomic region was performed by the Sanger platform. Details are found in the Supplementary Information (SI).

HBV and HDV genotypes/subgenotypes were determined by a phylogenetic tree constructed by the neighbor-joining method on MEGA6 software v6.0. The reference sequences for HDV genotypes/subgenotypes were retrieved from [16].

HDV-RNA sequencing was possible only for 41 out of 192 CHD group samples involved in the study and was performed at the virology laboratory of the University of Rome Tor Vergata. HBV-DNA sequencing was possible only for 24 out of 192 CHD group and for 91 out of 124 CHB group samples involved in the study, and it was performed at the virology laboratory of the University of Rome Tor Vergata.

### 2.4. Statistical Analysis

Statistical analysis was performed using IBM SPSS Statistics (v 23.0). The Mann–Whitney test was used for continuous data, while the chi-squared test was used for qualitative data in order to assess statistically significant differences between the different groups analyzed. Correlations with *p* < 0.05 were considered statistically significant.

The correlations of HBsAg isoforms with virological and biochemical parameters were assessed by the Spearman Rho test.

The levels of HBsAg isoforms were determined in the overall population of 192 CHD and 124 CHB patients and in the subset of 130 CHD and 37 CHB patients with total HBsAg > 2000 IU/mL as well as in the subset of 91 CHD and 23 CHB patients with total HBsAg > 5000 IU/mL (corresponding to the median and 75th percentile of total HBsAg in CHB patients, respectively). Moreover, the levels of HBsAg isoforms were determined by stratifying the 192 CHD patients according to HDV-RNA levels < or >1000 IU/mL, HBcrAg < or >3 logU/mL, and ALT < or >upper limit of normal (ULN), defined according to sex (35 U/L for males and 25 U/L for females) [17]. HDV-RNA > 1000 IU/mL has been proposed as a cut-off associated with an unfavorable clinical outcome in terms of increased risk of cirrhosis, transplantation, and death in recent studies [18,19]. The HBcrAg level of 3 logU/mL is the limit of quantification of the assay for HBcrAg quantification. Statistically significant differences were assessed by the Mann–Whitney test.

In the overall population, uni- and multivariable logistic regression analyses were performed to assess factors significantly associated with S-HBsAg > 2500 ng/mL, M-HBsAg > 400 ng/mL, and L-HBsAg > 1 ng/mL, considering the following variables: patients’ demographics, HBV-DNA, ALT, NUC treatment, HDV co-infection, and cirrhosis. Only patients with available data were considered in the analyses. The above-mentioned cut-offs of HBsAg isoforms were based on the median values of HBsAg isoforms in the overall population of 316 HBeAg-negative patients.

In patients with CHD, uni- and multivariable logistic regression analyses were performed to assess factors significantly associated with S-HBsAg > 3500 ng/mL, M-HBsAg > 800 ng/mL, and L-HBsAg > 1 ng/mL, considering the following variables: patients’ demographics, HBV-DNA > 20 IU/mL (representing the limit of quantification of the commercial assays used for HBV-DNA quantification in this study), NUC treatment, HDV-RNA > 1000 IU/mL, and cirrhosis. Only patients with available data were considered in the analyses. The above-mentioned cut-offs of HBsAg isoforms were based on the median values of HBsAg isoforms in the population with CHD (*N* = 192).

In all logistic regression analyses, after stepwise elimination for optimized Akaike information criterion, only variables showing a *p*-value < 0.05 in univariable analysis were included in multivariable analysis.

## 3. Results

### 3.1. Characteristics of Patients with CHD and CHB

Individuals involved in this study were all HBeAg-negative with comparable demographic characteristics and percentage of NUC treatment (Table 1). CHD was characterized by significantly lower HBV-DNA, lower platelet count, higher transaminases, and higher total HBsAg levels (Table 1). Total HBsAg was significantly higher among the CHD than the CHB group (Table 1). Median (IQR) HDV-RNA was 5.3 (3.8–6.1) logIU/mL (Table 1). A higher percentage of patients with cirrhosis was observed in CHD than in CHB (Table 1). In patients with CHD, different HDV subgenotypes were detected, with 1e being the most prevalent (65.9%), followed by 1c (26.9%), 1b (4.8%), and 1a (2.4%) (Table 1).

### 3.2. Levels of Total HBsAg and HBsAg Isoforms in Patients with CHD and CHB

**Figure 1:**
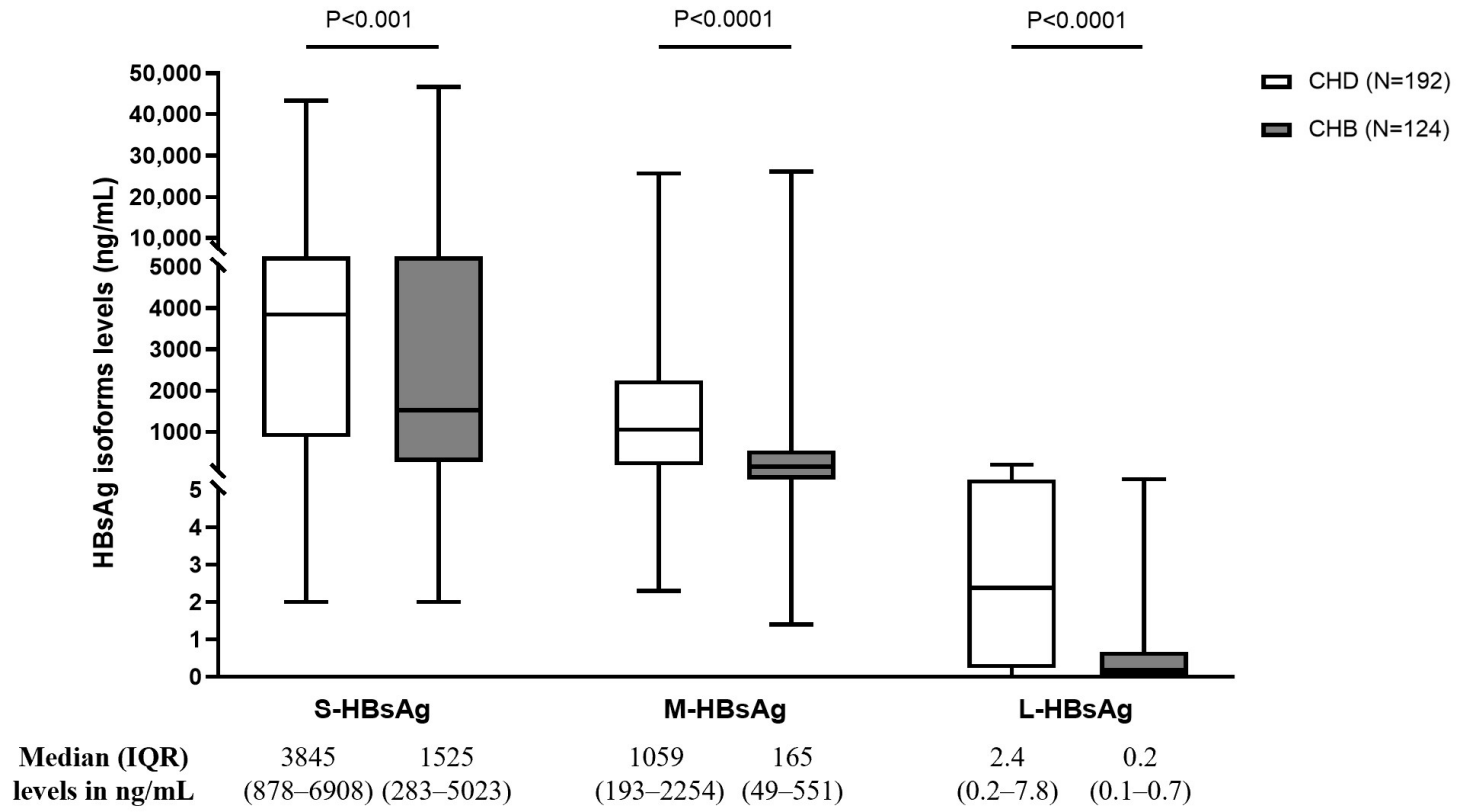
Box plots report the median, inter-quartile range, min, and max values of HBsAg isoforms in the overall population according to HDV co-infection status. Statistically significant differences were assessed by the Mann–Whitney test.

Patients with CHD were characterized by significantly higher levels of total HBsAg than those with CHB (median [IQR]: 5011 [1262–9945] vs. 2154 [870–5751] IU/mL by commercial assays, *p* = 0.009; 4746 [919–8848] vs. 1520 [256–5388] ng/mL by ELISA assay, *p* < 0.0001). Similarly, patients with CHD were characterized by significantly higher levels of all three HBsAg isoforms than those with CHB (Figure 1), with median (IQR) values in ng/mL of 3845 (878–6908) vs. 1525 (283–5023) for S-HBsAg, 1059 (193–2254) vs. 165 (49–551) for M-HBsAg and 2.4 (0.2–7.8) vs. 0.2 (0.1–0.7) for L-HBsAg (*p* < 0.001 for all comparisons) (Figure 1). Multivariable analysis confirmed that CHD is an independent factor significantly associated with higher levels of each HBsAg isoform (*p* < 0.0001 for all), after adjusting for HBV-DNA levels, NUC treatment, NUC duration, and cirrhosis status (Table 2). Conversely, an independent negative correlation was revealed for age (*p* < 0.001 for all) (Table 2). Moreover, comparable levels of HBsAg isoforms were observed in patients with HDV subgenotype 1c and 1e (*p* > 0.2 for all).

Finally, the levels of each HBsAg isoform were evaluated in the subset of CHD and CHB patients with total HBsAg > 2000 and >5000 IU/mL, corresponding to the median and the 75th percentile of total HBsAg observed in CHB patients. Notably, the levels of M- and L-HBsAg were significantly higher in CHD than in CHB patients (median [IQR] levels of 1678 [748–2763] vs. 510 [247–1309] ng/mL for M-HBsAg and 4.7 [1.7–10.1] vs. 0.5 [0.2–1.9] ng/mL for L-HBsAg using an HBsAg cut-off of 2000 IU/mL, *p* < 0.0001 for both; median [IQR] levels of 2164 [1237–3015] vs. 738 [437–1974] ng/mL for M-HBsAg and 5.7 [2.3–12.6] vs. 1.4 [0.5–8.6] ng/mL for L-HBsAg using an HBsAg cut-off of 5000 IU/mL, *p* < 0.01 for both). No significant difference was observed for S-HBsAg. This result suggests a specific association of HDV infection with these two HBsAg isoforms.

### 3.3. Correlation of HBsAg Isoforms with Virological and Biochemical Parameters in CHD

Each HBsAg isoform strongly correlated with total HBsAg measured by commercial assays (Rho = 0.89, 0.82, and 0.66 for S-HBsAg, M-HBsAg, and L-HBsAg, respectively; *p* < 0.0001 for all) (Figure S1). As expected, total HBsAg quantified by the ad hoc-designed ELISA assay showed a very strong correlation with total HBsAg by commercial assays (Rho = 0.92, *p* < 0.0001) (Figure S1).

**Figure 2:**
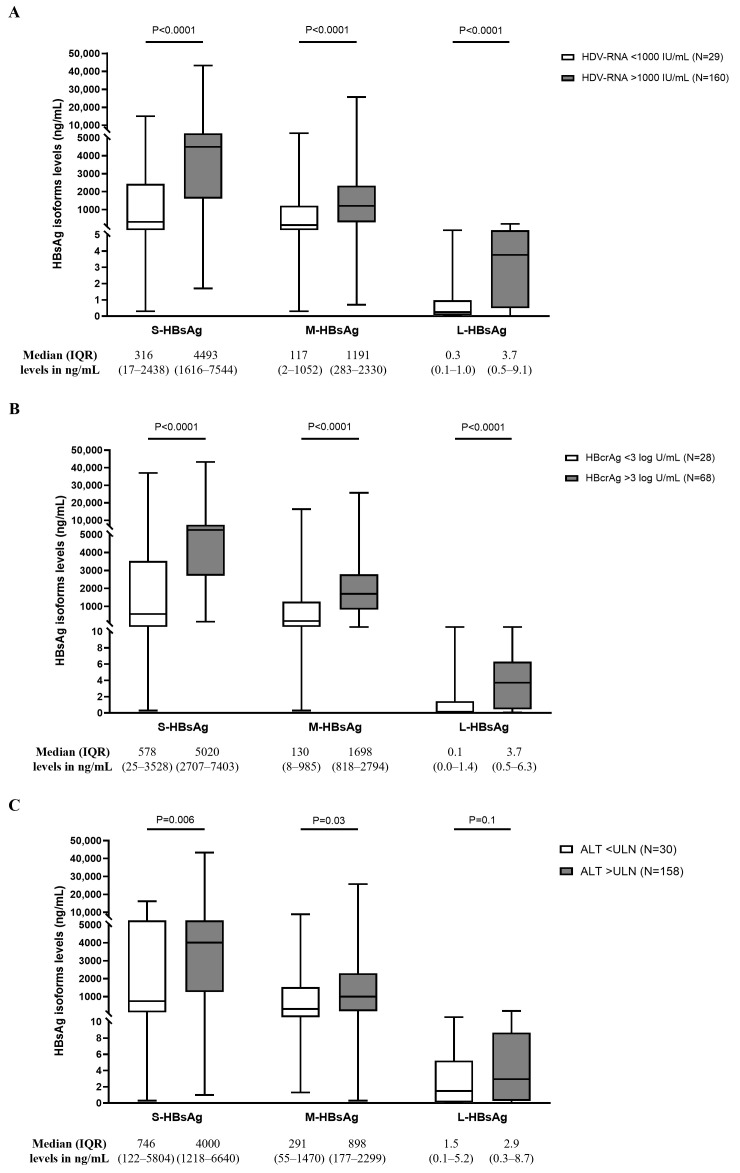
Box plots report the median, inter-quartile range, min, and max values of HBsAg isoforms in CHD patients according to HDV-RNA < or >1000 IU/mL (**A**), HBcrAg < or >3 logU/L (**B**), and ALT < or >40 U/L (**C**), respectively. Statistically significant differences were assessed by the Mann–Whitney test.

Furthermore, total HBsAg and each HBsAg isoform showed positive correlation with HDV-RNA (Rho = 0.54, 0.48, 0.45, and 0.44 for total HBsAg, S-HBsAg, M-HBsAg, and L-HBsAg, respectively; *p* < 0.0001 for all). This result was confirmed by stratifying patients with CHD according to HDV-RNA levels. Patients with HDV-RNA > 1000 IU/mL were characterized by significantly higher levels of all HBsAg isoforms than patients with HDV-RNA < 1000 IU/mL (Figure 2A). Multivariable analysis confirmed HDV-RNA > 1000 IU/mL as an independent factor significantly associated with higher levels of all HBsAg isoforms (Table 3). Conversely, no correlation was observed between HBsAg isoforms and HBV-DNA (analysis led in NUC-naïve patients) (*p* > 0.1 for all). Furthermore, by stratifying CHD patients according to HBV-DNA positivity, the levels of each HBsAg isoform were comparable between patients with HBV-DNA < 20 IU/mL and those with HBV-DNA > 20 IU/mL (median [IQR]: 3008 [785–6183] vs. 5010 [672–7442] ng/mL for S-HBsAg, 729 [119–2095] vs. 1428 [201–2442] ng/mL for M-HBsAg and 3.2 [0.3–9.1] vs. 2.4 [0.3–5.4] ng/mL for L-HBsAg; *p* > 0.05 for all comparisons).

HBsAg isoforms also showed a positive correlation with HBcrAg (Rho = 0.45, 0.47, and 0.42 for S-HBsAg, M-HBsAg, and L-HBsAg, respectively; *p* < 0.0001 for all). HBcrAg was also positively correlated with HDV-RNA (Rho = 0.37; *p* < 0.0001), while no correlation was noted with HBV-DNA (Rho = 0.11, *p* = 0.32). Patients with HBcrAg > 3 logU/mL showed significantly higher levels of all HBsAg isoforms than patients with HBcrAg < 3 logU/mL (*p* < 0.0001 for all) (Figure 2B). Similarly, HDV-RNA was higher in patients with HBcrAg > 3 logU/mL (Figure S2). This result was confirmed when the analysis was focused on CHD patients with HBV-DNA< 20 IU/mL (with limited/absent release of HBV mature virions).

Finally, the relation between HBsAg isoforms and ALT was evaluated. Patients with ALT > ULN were characterized by higher levels of S-HBsAg and M-HBsAg than patients with normal ALT (Figure 2C). Conversely, no relation between HBsAg isoforms and ALT was observed in CHB (median [IQR] levels in patients with ALT < ULN vs. patients with ALT > ULN: 759 [230–3190] vs. 1039 [90–5206] ng/mL for S-HBsAg, 112 [29–375] vs. 85 [20–419] ng/mL for M-HBsAg and 0.2 [0.1–0.7] vs. 0.1 [0.05–0.5] ng/mL for L-HBsAg; *p* > 0.5 for all comparisons).

No correlation of HBsAg isoforms with other biochemical/clinical parameters was noted.

## 4. Discussion

This is the first study that has systematically evaluated the correlation of HBsAg isoforms with virological and biochemical markers in a large set of patients with CHD. With respect to a set of HBsAg-positive patients with comparable HBeAg-negative status, demographics, and percentage of NUC treatment, CHD patients are characterized by a marked elevation not only of total HBsAg levels but also of all HBsAg isoforms. Multivariable analysis confirms the independent association of CHD with increased levels of HBsAg isoforms after correction for NUC treatment, NUC duration, ALT levels, and cirrhosis status, suggesting that CHD may be associated with an enhanced production of HBV surface glycoproteins. Multivariable analysis also showed the independent association of age with lower levels of HBsAg isoforms. This result is in line with previous studies, showing that HBsAg levels undergo a progressive decrease with increasing patients’ age, as a consequence of hepatocyte turnover and reduction of viral reservoir in both CHD and CHB patients [20,21].

In patients with CHD, we also noted positive correlations of HBsAg isoforms with HDV-RNA, but not with HBV-DNA, potentially suggesting a preferential recruitment of HBsAg isoforms in HDV virions. However, the fact that these correlations are not particularly strong (Rho ranging from 0.44 to 0.54) suggests that other factors, such as the release of subviral particles, can contribute to the overall burden of HBsAg isoforms. Unravelling this issue in in vitro studies can be critical to understand HDV’s capability to perturb the secretion of HBV particles, thus promoting the release of HDV virions.

Our study showed for the first time the positive association of HBsAg isoforms with HBcrAg in patients with CHD. In line with a previous study [22], a positive association was also noted between HBcrAg and HDV-RNA but not with HBV-DNA. In the setting of HBeAg negativity, HBcrAg quantification reflects the amount of the capsid and truncated p22 proteins present in mature HBV virions, empty virions, and naked capsids (known to be released from the infected hepatocytes and to contain immature single-stranded DNA and pregenomic RNA). Although multiple hypotheses may be formulated, it is plausible that the preferential recruitment of HBsAg isoforms in HDV virions may reduce the envelopment of HBV capsids, thus potentially favoring the release of naked capsids. Although further in vitro studies are necessary, this can shed light on a new interpretation of HBcrAg levels in the setting of HBeAg-negative CHD. This reinforces the importance of unraveling the interplay between HBV and HDV to dissect the pathogenic mechanisms underlying CHD.

Overall, our findings can pave the way for further studies aimed at deepening the role of HBsAg isoforms in predicting different clinical phenotypes and liver disease progression. In this regard, we have found that CHD patients with ALT > ULN are characterized by higher levels of S-HBsAg and M-HBsAg (association not observed in patients with CHB). This result can be explained by the fact that HDV itself is known to be a potent inducer of liver inflammation [23]. At the same time, since both S-HBsAg and M-HBsAg are known to be highly immunogenic and to contain B- and T-cell epitopes [5,24], the potential contribution of these two HBsAg isoforms in supporting HDV-induced cytolytic activity cannot be completely excluded. Furthermore, previous studies have highlighted the association of more elevated levels of M-HBsAg with an unfavorable clinical outcome [9,10]. In particular, a previous study by our group showed that an increase in M-HBsAg levels characterizes a significant fraction of cirrhotic patients developing HCC despite prolonged HBV suppression, suggesting a potential role of M-HBsAg kinetics in identifying patients at higher HCC risk [9]. In a similar direction, another study showed that M-HBsAg levels tended to be higher in patients who developed decompensation during follow-up, suggesting a role as a prognosis marker [10]. Overall, these results support that drugs interfering with HBsAg production/release can play a key role in therapeutic strategies aimed at curing HDV infection. In particular, two novel classes of anti-HDV drugs are under clinical evaluation: the nucleic acid polymers (specifically binding to the HBsAg domain present in all three HBsAg isoforms) and the small interfering RNAs (specifically binding to the mRNA encoding HBsAg isoforms). These novel therapeutic strategies have proven not only to reduce HDV replicative activity but also to dampen liver inflammation, presenting a good safety profile [25,26].

Beyond clinical outcome, the role of HBsAg isoforms has been explored in predicting treatment response. In particular, a recent study by our group has identified specific cut-off values of HBsAg isoforms capable of predicting the achievement of HDV-RNA undetectability with or without ALT normalization after 48 and 96 weeks of bulevirtide [11].

Furthermore, it should be noted that the quantification of HBsAg isoforms assays is based on the cheap and easy-to-use sandwich ELISA technology with a low turnaround time, supporting its feasibility in clinical practice. On this basis, although further studies are necessary, HBsAg isoform quantification could represent a valid and affordable alternative to characterize virological profiles of CHD patients and/or to monitor treatment response. This could be particularly relevant in low-/middle-income settings, where access to HDV-RNA quantification by real-time PCR is still lacking due to the high costs.

It should be noted that this study has some limitations: (i) the study was not designed to be a case–control study; (ii) HDV-RNA quantification was also carried out using a home-made and non-standardized assay.

In conclusion, CHD is characterized by a more elevated production of total HBsAg and all three HBsAg isoforms, paralleling HDV-RNA and HBcrAg production. This reinforces the need to better elucidate the interplay between HBV and HDV. The association of HBsAg isoforms with elevated ALT in CHD suggests their potential contribution in supporting HDV-induced pro-inflammatory stimuli. Their role in identifying patients at higher risk of disease progression deserves further investigation.

## Figures and Tables

**Table 1:** Characteristics of the study population.

Variables ^a^	CHD (*N* = 192)	CHB (*N* = 124)	*p*-Value ^b^
Age, years	53 (43–60)	50 (39–62)	0.24
Male sex	117 (60.9)	80 (64.5)	0.55
Country of origin			
*Italy*	103 (53.6)	70 (56.5)	0.08
*Eastern Europe*	81 (42.2)	42 (33.8)	
*Other*	8 (4.2)	12 (9.7)	
NUC treatment	116 (64.4) ^c^	80 (64.5)	0.99
NUC duration, years	5 (2–8)	4 (2–6)	0.80
ALT > ULN ^d^	162 (84.4)	30 (31.6) ^e^	<0.0001
ALT, U/L	80 (51–133)	30 (20–47)	**<0.0001**
AST, U/L	62 (42–99)	26 (17–28)	**<0.0001**
Platelets, units ×10^3^/mm^3^	130 (83–164)	222 (193–287)	**<0.0001**
Cirrhosis	127 (74.7) ^f^	10 (13.5) ^f^	**<0.0001**
HCC	12 (7.5) ^g^	4 (4.2) ^g^	0.43
HDV-RNA, log IU/mL	5.3 (3.8–6.1)	-	-
HDV-RNA < 1000 IU/mL	29 (15.2) ^h^	-	-
HDV genotype/subgenotype ^i^			
*1a*	1 (2.4)	-	-
*1b*	2 (4.8)	-	
*1c*	11 (26.9)	-	
*1e*	27 (65.9)	-	
HBV-DNA, log IU/mL	1.0 (0.0–1.9)	2.6 (1.3–3.5)	**<0.0001**
HBV-DNA < 20 IU/mL	123 (64.1)	31 (33.3) ^j^	**<0.0001**
HBV genotype ^k^			
*A*	1 (4.2)	16 (17.6)	0.14
*D*	22 (91.6)	66 (72.5)	
*E*	1 (4.2)	9 (9.9)	

^a^ Data for continuous variables are reported as median (IQR), while data for qualitative variables are reported as *N* (%). ^b^ *p*-values were calculated by the Mann–Whitney test for continuous variables and by the chi-squared test for qualitative variables. Statistically significant differences (*p* < 0.05) are reported in bold. ^c^ Percentages calculated on 180 patients with data available. ^d^ ULN was defined as >35 U/L for males and >25 U/L for females [17]. ^e^ Percentage calculated on 95 patients with data available. ^f^ Percentages calculated on 170 CHD and 74 CHB patients with data available. ^g^ Percentages calculated on 160 CHD and 96 CHB patients with data available. ^h^ All 29 individuals have detectable HDV-RNA. ^i^ Percentages calculated on 41 patients with data available. ^j^ Percentage calculated on 93 patients with data available. ^k^ Percentages calculated on 24 CHD and 91 CHB patients with data available. Abbreviations: NUC, nucleos(t)ide analogs; ALT, alanine transaminase; ULN, upper limit of normal; HCC, hepatocellular carcinoma.

**Table 2:** Multivariable logistic regression analyses to identify factors significantly correlated with higher levels of HBsAg isoforms in the overall population.

	S-HBsAg > 2500 ng/mL ^a^				M-HBsAg > 400 ng/mL ^a^				L-HBsAg > 1 ng/mL ^a^			
Variables	Univariable Analysis		Multivariable Analysis		Univariable Analysis		Multivariable Analysis		Univariable Analysis		Multivariable Analysis	
	Crude OR (95% CI)	*p*-Value	Adjusted OR (95% CI)	*p*-Value	Crude OR (95% CI)	*p*-Value	Adjusted OR (95% CI)	*p*-Value	Crude OR (95% CI)	*p*-Value	Adjusted OR (95% CI)	*p*-Value
**Age,** **per year increase**	**0.96 (0.94–0.98)**	**<0.0001**	**0.95 (0.94–0.97)**	**<0.0001**	**0.98 (0.96–0.99)**	**0.009**	**0.97 (0.94–0.99)**	**0.001**	**0.98 (0.96–0.99)**	**0.02**	**0.96 (0.94–0.98)**	**0.001**
**Sex,** **male vs. female ^b^**	0.97 (0.61–1.52)	0.88			1.01 (0.63–1.58)	0.99			0.74 (0.47–1.17)	0.19		
**HBV-DNA,** **per log IU/mL increase ^c^**	0.97 (0.91–1.02)	0.21			0.96 (0.91–1.01)	0.15			0.97 (0.92–1.02)	0.25		
**ALT,** **per U/L increase ^d^**	1.0 (0.99–1.01)	0.57			1.0 (0.99–1.01)	0.21			1.0 (0.99–1.01)	0.53		
**NUC treatment,** **yes vs. no ^b,e^**	0.67 (0.41–1.08)	0.11			0.67 (0.41–1.08)	0.11			1.22 (0.75–1.98)	0.43		
**NUC duration,** **per year increase ^e^**	0.96 (0.88–1.06)	0.43			1.03 (0.94–1.13)	0.55			1.01 (0.92–1.1)	0.89		
**Chronic HDV infection,** **yes vs. no ^b^**	**2.49 (1.56–3.97)**	**<0.0001**	**3.01 (1.81–4.96)**	**<0.0001**	**3.67 (2.27–5.94)**	**<0.0001**	**3.73 (1.83–7.62)**	**<0.0001**	**7.16 (4.18–12.27)**	**<0.0001**	**4.57 (2.18–9.58)**	**<0.0001**
**Cirrhosis,** **yes vs. no ^b,f^**	1.39 (0.84–2.32)	0.19			**1.97 (1.18–3.28)**	**0.009**	1.23 (0.64–2.38)	0.54	**2.73 (1.63–4.59)**	**<0.0001**	1.72 (0.88–3.35)	0.11

The table reports the uni- and multivariate analyses of factors associated with higher levels of all HBsAg isoforms. Variables with a *p*-value < 0.05 in univariate analysis were included in multivariate analysis. Statistically significant *p*-values are reported in bold. ^a^ These values represent the median levels of the three HBsAg isoforms in the overall population (*N* = 316). ^b^ Reference group. ^c^ Uni- and multivariable analyses were performed on 285 patients with available data. ^d^ Uni- and multivariable analyses were performed on 287 patients with available data. ^e^ Uni- and multivariable analyses were performed on 304 patients with available data. ^f^ Uni- and multivariable analyses were performed on 244 patients with available data. Abbreviations: OR: odds ratio; CI, confidence interval; ALT, alanine transaminase; NUC, nucleos(t)ide analogs; S-HBsAg, small hepatitis B surface antigen; M-HBsAg, middle hepatitis B surface antigen; L-HBsAg, large hepatitis B surface antigen.

**Table 3:** Multivariable logistic regression analyses to identify factors significantly correlated with higher levels of HBsAg isoforms in CHD patients.

	S-HBs > 3500 ng/mL ^a^				M-HBs > 800 ng/mL ^a^				L-HBs > 3 ng/mL ^a^			
Variables	Univariable Analysis		Multivariable Analysis		Univariable Analysis		Multivariable Analysis		Univariable Analysis		Multivariable Analysis	
	Crude OR (95% CI)	*p*-Value	Adjusted OR (95% CI)	*p*-Value	Crude OR (95% CI)	*p*-Value	Adjusted OR (95% CI)	*p*-Value	Crude OR (95% CI)	*p*-Value	Adjusted OR (95% CI)	*p*-Value
**Age,** **per year increase**	**0.96 (0.94–0.99)**	**0.004**	**0.96 (0.94–0.99)**	**0.007**	**0.96 (0.94–0.99)**	**0.002**	**0.96 (0.93–0.99)**	**0.01**	**0.97 (0.95–0.99)**	**0.03**	**0.97 (0.94–0.99)**	**0.03**
**Sex,** **male vs. female ^b^**	0.74 (0.41–1.32)	0.31			0.86 (0.48–1.54)	0.61			0.76 (0.43–1.37)	0.37		
**HBV-DNA > 20 IU/mL,** **yes vs. no ^b^**	1.81 (0.98–3.32)	0.06			1.73 (0.94–3.19)	0.08			0.79 (0.43–1.44)	0.44		
**ALT > ULN,** **yes vs. no ^b^**	**2.65 (1.14–6.14)**	**0.02**	1.26 (0.45–3.51)	0.66	1.96 (0.88–4.39)	0.11			1.73 (0.77–3.87)	0.18		
**NUC treatment,** **yes vs. no ^b,c^**	0.81 (0.43–1.48)	0.48			**0.51 (0.27–0.94)**	**0.03**	**0.51 (0.26–0.98)**	**0.05**	**1.85 (0.99–3.45)**	**0.05**	1.86 (0.95–3.63)	0.07
**HDV-RNA > 1000 IU/mL,** **yes vs. no ^b^**	**4.81 (1.86–12.44)**	**0.001**	**4.81 (1.82–12.66)**	**0.002**	**3.38 (1.41–8.07)**	**0.006**	**2.67 (1.01–7.08)**	**0.05**	**7.45 (2.48–22.39)**	**<0.0001**	**7.02 (1.96–25.14)**	**0.003**
**Cirrhosis,** **yes vs. no ^b,d^**	0.86 (0.43–1.71)	0.66			0.51 (0.25–1.05)	0.07			1.46 (0.72–2.93)	0.29		

The table reports the uni- and multivariable analyses of factors associated with higher levels of all HBsAg isoforms. Variables with a *p*-value < 0.05 in univariable analysis were included in multivariable analysis. Statistically significant *p*-values are reported in bold. ^a^ These values represent the median levels of the three HBsAg isoforms in the CHD population (*N* = 192). ^b^ Reference group. ^c^ Uni- and multivariable analyses were performed on 180 CHD patients with available data. ^d^ Uni- and multivariable analyses were performed on 170 CHD patients with available data. Abbreviations: OR: odds ratio; CI, confidence interval; S-HBs, small hepatitis B surface antigen; M-HBs, middle hepatitis B surface antigen; L-HBs, large hepatitis B surface antigen; ALT, alanine transaminase; ULN, upper limit of normal; NUC, nucleos(t)ide analogs.

## Data Availability

Research data supporting reported results of this manuscript are available upon request to the corresponding authors.

## Supplementary Materials

The following supporting information can be downloaded at: https://www.mdpi.com/article/10.3390/v18050515/s1, Figure S1: The graphs report the positive correlations of total HBsAg quantified by commercial assays with total HBsAg and each HBsAg isoform quantified by ad hoc-designed ELISA assays in CHD. Statistically significant correlations were assessed by the Spearman Rho test; Figure S2: Box plots report the median, inter-quartile range, min, and max values of HDV-RNA according to HBcrAg < or >3 log U/mL. Statistically significant differences were assessed by the Mann–Whitney test.
