# Supplementary material for: Chronic HDV Infection Shows Higher HBsAg Isoform Levels than HBV Infection, Paralleling HDV Replicative Activity
Source: Viruses. 2026 Apr 30;18(5):515. doi: 10.3390/v18050515 (PMC13211560; doi:10.3390/v18050515)
Supplement: Supplementary file 1 [file viruses-18-00515-s001.zip › Supplementary_Information_revised.pdf]

Supplementary Information

Methods

HDV-RNA extraction, reverse-transcription, amplification and sequencing

In order to sequence HDV-RNA, viral nucleic acid was extracted from 140µL of plasma samples by using the the commercial QIAmp Viral RNA Mini kit (Qiagen, #52904) for manual extraction of viral RNA. After extraction, RNA was denatured at 94°C for 3 minutes and then quickly transferred to -80°C for 5 minutes. After thermal shock, cDNA was generated by using SuperScript™ IV (Invitrogen by Life Technologies) with the following protocol: 60°C for 20 minutes and 80°C for 10 minutes. The specified nucleotide region was then amplified by using KAPA HiFi ReadyMix (Roche, #07958935001) and the following primer pairs: HDV_full_F-5' TGGAGATGCCATGCCGACCCGAA-3' and HDV_full_R-5'-TCCTCGCGGTCCGACCTGGGCAT-3'. PCR conditions were: 95°C for 5min, 40 cycles (98°C 20s, 70°C 15s, 72°C 2:00min), and final extension at 72°C for 5min.

Amplified PCR-products were purified by AmpureXP magnetic beads (Beckman Coulter, USA), Massachusetts, US) and the library was generated by using Nextera XT DNA Library Preparation Kit (Illumina, USA). Sequencing was performed on Illumina MiSeq platform by using MiSeq Reagent Kit v2 500 cycles. HDV-full genome (MH457154.1) was used as reference sequence for the bioinformatic analysis. Once extrapolated by FastQ, HDV sequences were aligned by MAFFT and genotype were attributed by phylogenetic tree constructed by Neighbor-Joining method on MEGA6 software. Branching order reliability was assessed by bootstrap analysis of 1000 replicates.

Consensus sequences for genotype determination were retrieved by [16].

HDV-RNA sequencing was possible only for 41 out of 192 CHD group samples involved in the study and was performed at the virology laboratory of University of Rome Tor Vergata.

HBV-DNA extraction, amplification and sequencing

In order to sequence HBV-DNA, viral nucleic acid was extracted from 400 uL plasma samples using QIAmp DNA mini-kit (Qiagen, Germantown, USA), and then the nucleotide region encoding HBsAg and overlapping reverse-transcriptase (RT) was amplified with Amplitaq-Gold polymerase using S-HBV_F1-5′GGTCACCATATTCTTGGGAA and S-HBV_R1-5′GTGGGGGTTGCGTCAGCAAA primer pairs for first-round PCR. PCR conditions were: 93°C for 12min, 40 cycles (94°C 50s, 57°C 50s, 72°C 90s), and 72°C for 10min. When necessary, a second-round PCR was performed, using S-HBV_F2-5′GGTGGACTTCTCTCAATTTT and S-HBV_R2-5'TGGCGAGAAAGTGAA primer pairs. Second-round PCR conditions were: 93°C for 12min, 40 cycles (94°C 50s, 55°C 50s, 72°C 80s), and 72°C for 10min.

PCR-products were then purified by ExoSAP-IT PCR Product Cleanup Reagent (ThermoFisher Scientific, Massachusetts, US) and sequenced by Sanger-based method using different sequence-specific primers and a BigDye-Terminator v.3.1 cycle sequencing kit (Applied-Biosystems, Foster City, USA) on the ABI Prism 3130XL (Applied-Biosystems, Foster City, USA). HBsAg/RT-sequences were analysed using SeqScape-v.2.6 software (Applied-Biosystems), then aligned using BioEdit 7.0 software (Hall, Nucleic Acid Res 1999). HBV-full genome (NC_003977.2) was used as reference sequence for the alignment.

HBV-DNA sequencing was possible only for 24 out of 192 CHD group and for 91 out of 124 CHB group samples involved in the study and was performed at the virology laboratory of University of Rome Tor Vergata.

Figure legends

Figure S1.

The graphs report the positive correlations of total HBsAg quantified by commercial assays with total HBsAg and each HBsAg isoform quantified by *ad hoc* designed ELISA assays in CHD. Statistically significant correlations were assessed by Spearman Rho test.

Figure S2.

Box plots report the median, inter-quartile range, min and max values of HDV-RNA according to HBcrAg < or > 3 log U/mL. Statistically significant differences were assessed by Mann-Whitney test.
