# Supplementary figures and images for: Chronic HDV Infection Shows Higher HBsAg Isoform Levels than HBV Infection, Paralleling HDV Replicative Activity
Source: Viruses. 2026 Apr 30;18(5):515. doi: 10.3390/v18050515 (PMC13211560; doi:10.3390/v18050515)

**Figure S1.**

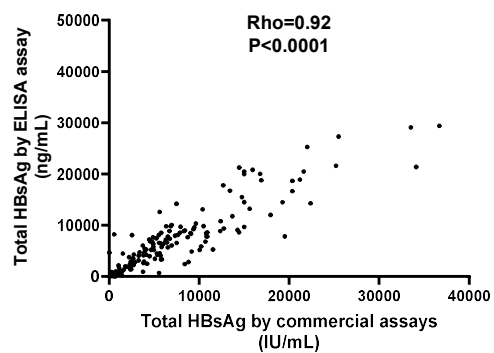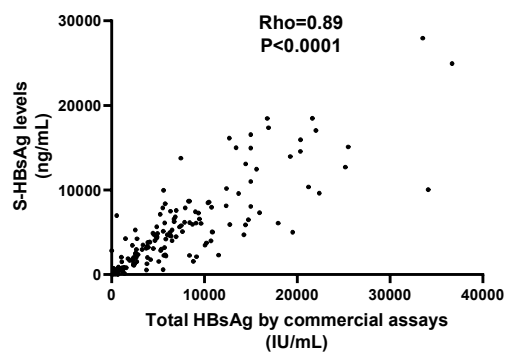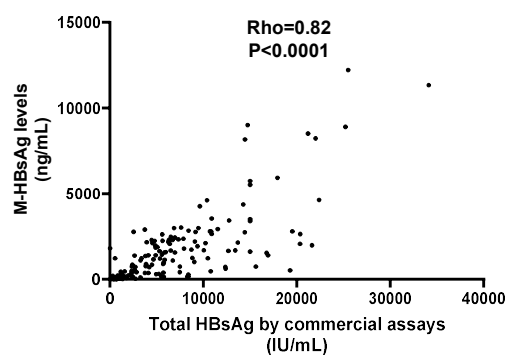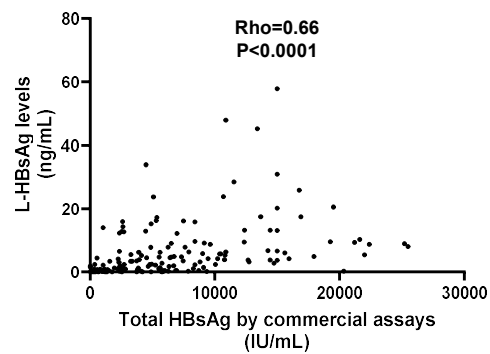

Figure S2.

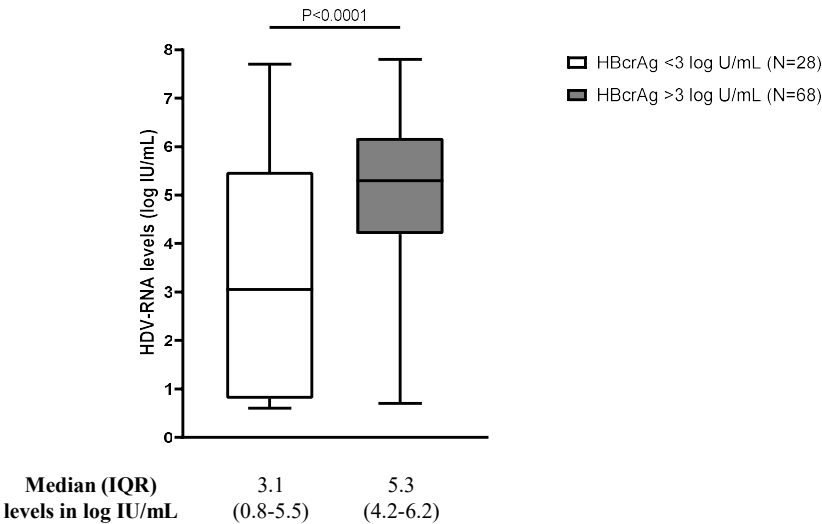

Supplement: Supplementary file 1 [file viruses-18-00515-s001.zip › Suppl Figures_revised.pdf]
